# Protein interaction networks at the host–microbe interface in *Diaphorina citri*, the insect vector of the citrus greening pathogen

**DOI:** 10.1098/rsos.160545

**Published:** 2017-02-08

**Authors:** J. S. Ramsey, J. D. Chavez, R. Johnson, S. Hosseinzadeh, J. E. Mahoney, J. P. Mohr, F. Robison, X. Zhong, D. G. Hall, M. MacCoss, J. Bruce, M. Cilia

**Affiliations:** 1 Robert W. Holley Center for Agriculture and Health, Emerging Pests and Pathogens Research Unit, USDA Agricultural Research Service, Ithaca, NY, USA; 2 Boyce Thompson Institute for Plant Research, Ithaca, NY, USA; 3 Department of Genome Sciences, University of Washington, Seattle, WA, USA; 4 Plant Pathology Department, Faculty of Agriculture, Tarbiat Modares University, Tehran, Iran; 5 US Horticultural Research Laboratory, Subtropical Insects and Horticulture Research Unit, USDA Agricultural Research Service, Ft. Pierce, FL, USA; 6 Plant Pathology and Plant-Microbe Biology Section, School of Integrative Plant Science, Cornell University, Ithaca, NY, USA

**Keywords:** citrus greening disease, ‘*Candidatus* Liberibacter asiaticus’, proteomics, endosymbiont, Asian citrus psyllid, insect vector

## Abstract

The Asian citrus psyllid (*Diaphorina citri)* is the insect vector responsible for the worldwide spread of ‘*Candidatus* Liberibacter asiaticus’ (CLas), the bacterial pathogen associated with citrus greening disease. Developmental changes in the insect vector impact pathogen transmission, such that *D. citri* transmission of CLas is more efficient when bacteria are acquired by nymphs when compared with adults. We hypothesize that expression changes in the *D. citri* immune system and commensal microbiota occur during development and regulate vector competency. In support of this hypothesis, more proteins, with greater fold changes, were differentially expressed in response to CLas in adults when compared with nymphs, including insect proteins involved in bacterial adhesion and immunity. Compared with nymphs, adult insects had a higher titre of CLas and the bacterial endosymbionts Wolbachia, Profftella and Carsonella. All Wolbachia and Profftella proteins differentially expressed between nymphs and adults are upregulated in adults, while most differentially expressed Carsonella proteins are upregulated in nymphs. Discovery of protein interaction networks has broad applicability to the study of host–microbe relationships. Using protein interaction reporter technology, a *D. citri* haemocyanin protein highly upregulated in response to CLas was found to physically interact with the CLas coenzyme A (CoA) biosynthesis enzyme phosphopantothenoylcysteine synthetase/decarboxylase. CLas pantothenate kinase, which catalyses the rate-limiting step of CoA biosynthesis, was found to interact with a *D. citri* myosin protein. Two Carsonella enzymes involved in histidine and tryptophan biosynthesis were found to physically interact with *D. citri* proteins. These co-evolved protein interaction networks at the host–microbe interface are highly specific targets for controlling the insect vector responsible for the spread of citrus greening.

## 1. Introduction

The Asian citrus psyllid (ACP, *Diaphorina citri*) is the primary insect vector of ‘*Candidatus* Liberibacter asiaticus’ (CLas), the bacterial pathogen associated with citrus greening disease [1,2]. Citrus greening is the most significant disease of citrus worldwide, with extreme outbreaks currently devastating production in Brazil, Florida and many other regions of the world. The vector's range has grown to include all citrus-growing states in the USA since its first documented appearance in Florida in 1998 [3]. The economic consequences of the spread of citrus greening disease throughout the state of Florida since the first reported case in 2005 have been disastrous: statewide citrus production dropped from 242 million boxes in 2005 to 104.6 million boxes in 2014 [4]. In 2012, the first infected tree was discovered in California, and in 2015 the California Department of Food and Agriculture announced that the disease had been detected in 10 additional trees. In spite of intensive eradication efforts, the ACP has spread extensively throughout Southern California, and novel disease control strategies are required to prevent the spread of the disease by this entrenched vector population.

Transmission of the citrus greening pathogen to a healthy tree depends on the ability of CLas to circulate within the insect vector. Vertical pathogen transmission from mother to offspring relies upon the plant host as an intermediary, although low rates of transovarial CLas transmission have also been reported [5]. Hemipteran insects, such as the ACP, inject saliva into their host plant during feeding. CLas is transmitted to a healthy citrus plant when pathogen-containing saliva is injected into a sieve tube element cell by the insect stylet, and as the pathogen replicates in the plant its distribution is restricted to the phloem [6]. The ACP lays its eggs on young flush tissue, and high levels of CLas accumulate locally as infected adults feed during oviposition. Young citrus flush tissue can become infectious as early as 15 days after psyllid inoculation [7], suggesting a model whereby systemic infection of the tree is not necessary for tree-to-tree spread. After emergence from eggs onto infected plants, ACP nymphs begin feeding from the phloem and are exposed to the bacterium, which crosses the first barrier to circulative transmission by acquisition into cells of the insect gut [8]. Acquisition of CLas by nymphs during feeding occurs at a much higher rate than during feeding by adults [9,10], suggesting that the response of the ACP to CLas depends on the insect developmental stage. Analysis of the impact of CLas on vector fitness reveals that CLas(+) ACP have higher fecundity than CLas(−) insects, although nymphal development rate and adult survival are reduced in CLas(+) colonies [11]. Previous proteomic analysis of CLas(+)/(−) adult ACP samples revealed that proteins associated with stress and defence responses are upregulated in CLas-exposed insects, although it is unclear whether these changes represent a direct response to exposure to the pathogen, or if the insect defence response is indirectly triggered by CLas-induced changes in plant quality [12].

CLas cells that cross the ACP gut barrier enter the haemolymph, the fluid that circulates in the open circulatory system of the insect, and must evade the cellular immune response [13] to arrive at the surface of the salivary glands [14]. Entry into and exit from the salivary glands constitute the final barriers to circulative pathogen transmission through the insect [8]. This circulative mode of transmission necessitates extensive contact between CLas and the ACP, and protein–protein interactions are likely to be central to cellular entry and haemolymph transit by the pathogen.

Protein–protein interactions are the functional interface of many biological processes, including interactions between hosts and microbes [15]. Advances in strategies to identify protein–protein interactions have included the development of specialized cross-linker compounds capable of penetrating live cells and forming covalent interactions between associated proteins [16,17]. A mass spectrometry-compatible protein cross-linker was used to identify protein interaction networks including ACP, CLas and endosymbiont proteins [18,19]. Three major bacterial endosymbionts associated with the ACP have been identified, with proposed functions in defence, nutritional supplementation and reproductive manipulation, respectively: ‘*Candidatus* Profftella armatura’ [20,21], ‘*Candidatus* Carsonella rudii’ [22] and Wolbachia [23,24]. Nutritional interactions between hemipteran insects and their microbial endosymbionts are critical to the insect's adaptation to a phloem sap diet, and metabolic interdependency between symbionts and the insect host has led to a dramatic reduction in symbiont genome size and specialization of metabolic capabilities [21,22,25]. Protein interactions between the ACP and endosymbiont amino acid biosynthesis proteins may be relevant to the nutritional dependency central to this symbiosis. With the increased prevalence of both the insect vector and the pathogen in nearly all citrus-growing regions of the world, development of specific and effective inhibitors to interfere with protein interactions between ACP, CLas and ACP endosymbionts represents a promising strategy to block the spread of citrus greening disease.

## 2. Material and methods

### 2.1. Insect rearing

Asian citrus psyllid colonies have been maintained for several years at the US Horticultural Research Laboratory (Ft. Pierce, FL, USA) on *Citrus medica* (citron) plants. ACP colonies are maintained in growth chambers with a 14 L : 10 D photoperiod, at 26°C and 70% humidity. The CLas infection rate of the ACP populations used for these studies was determined by calculating the percentage of insects testing qPCR positive in each colony; this rate was 90% (27/30), 80% (24/30) and 53% (16/30) for the three colonies used in this study. Adult ACP collected from CLas(+)/(−) colonies were transferred onto healthy and CLas-infected citron plants and allowed to oviposit. Adults were removed after the 2-day oviposition period and discarded, and fifth instar nymphs were collected after approximately three weeks. Nymphs that remained on experimental plants completed development into adults, which were collected within one week of metamorphosis.

### 2.2. Microbial copy number quantification by qPCR

Insects were flash frozen and cryoground (Retsch Mixer Miller MM400), and DNA was extracted using the DNeasy Blood and Tissue Kit (Qiagen). The Applied Biosystems 7900HT instrument was used for qPCR analysis. CLas qPCR was performed using the Taqman Universal qPCR Master Mix (Life Technologies) and primer and probe sequences from [26]. Taqman qPCR conditions are: 10 min at 95°C; 40 cycles of (15 s at 95°C; 60 s at 60°C). Endosymbiont qPCR was performed using the Fast SYBR Green Master Mix (Life Technologies) and primer sequences from [27]. SYBR Green qPCR conditions are: 20 s at 95°C; 40 cycles of (3 s at 95°C; 30 s at 60°C). Absolute quantification of microbial copy number was enabled by comparing Ct values from biological samples to Ct values from standard curves made using serial dilutions of synthetic plasmids containing qPCR target region.

### 2.3. Digital droplet PCR analysis of Asian citrus psyllid gene expression

ACP (10 insects per sample) were collected from citrus plants, flash frozen and cryoground (Retsch Mixer Mill 400). RNA extraction was performed using the RNeasy kit (Qiagen). Genomic DNA was removed by treatment of samples with RNase-free DNase I (Thermo Fisher), and RNA was quantified by Nanodrop (Thermo Fisher). RNA (1 µg) was used for cDNA synthesis with a haemocyanin 1 gene-specific primer (5′AGTAGGAGCCTCGTCTGATG). Digital droplet PCR (ddPCR) was performed on the QX100 ddPCR system (Bio-Rad) using TaqMan probe with FAM dye and ZEN-Iowa Black Fluorescent quencher (5′FAM-AGCCTTCCA/ZEN/ACATCGCCTGAACCA/IABkFQ) and primers (Forward: GACATGAATACCATGAACAACAGG; Reverse: AACTTGGGTCCAATGAATACTCTC) specifically targeting haemocyanin 1. ddPCR reactions were assembled with cDNA, primers, probe and ddPCR Supermix for Probes (no dUTP, Bio-Rad) and droplets were generated using the manufacturer's protocol with the QX100 Droplet Generator machine and the generated droplets were transferred into a 96-well PCR plate (twin.tec PCR Plate 96, unskirted, clear, Eppendorf). Plates were sealed by foil and a PCR reaction under the following conditions was performed on a C1000 Touch Thermal Cycler (Bio-Rad): 10 min at 95°C, 40 cycles (30 s at 94°C, 60 s at 60°C), 98°C for 10 min. FAM fluorescence in the generated droplets was measured using the QX100 Droplet Reader machine and QuantaSoft Software (Bio-Rad). Three technical replicate ddPCR reactions were performed for each biological sample.

### 2.4. Quantitative mass spectrometry-based proteomics

ACP peptide samples for liquid chromatography mass spectrometry (LC/MS) analysis were prepared and analysed following the sample preparation, LC/MS and data analysis protocols detailed in [12]. For each biological replicate, 75 adult or nymph ACP were flash frozen and used as starting material for whole insect protein extraction, and 200 live adult or nymph ACP were used for Percoll gradient fractionation. Statistical analysis of weighted spectral count data for identification of proteins differentially abundant between sample categories was performed in Scaffold (v. 4.6.1, Proteome Software Inc., Portland, OR, USA) with Fisher's exact test (significance level *p* < 0.05 using the Hochberg–Benjamini multiple test correction).

### 2.5. Protein interaction reporter cross-linking

Percoll density gradient centrifugation was used to isolate from approximately 1000 CLas(+) ACP a cellular fraction enriched for intact microbial cells [12]. Details of the cross-linking and analysis protocols are provided in electronic supplementary material, file S1.

### 2.6. Protein structure modelling

Phyre2 [28] was used for homology modelling of protein three-dimensional structures, and RasMol [29] was used to visualize .pdb files created by Phyre2 and to map the location of specific amino acid residues in the predicted three-dimensional structure.

## 3. Results

### 3.1. Effect of developmental stage and *Candidatus* Liberibacter asiaticus infection on protein expression in the Asian citrus psyllid

Peptides from a total of 4186 proteins were identified in whole insect samples using mass spectrometry. Peptide and protein datasets and mass spectrometry data are available at ProteomeXchange [30] (accession numbers given in the Data accessibility section). These data, resulting from analysis of three biological replicates each of nymph and adult ACP populations collected from both healthy and CLas-infected citrus, represent significant advances from previous proteomic studies of the ACP [12,31] and the most comprehensive proteomic coverage for the ACP to date.

The impact of CLas on protein expression was more dramatic in adult insects than in nymphs. A total of 356 proteins were differentially expressed between CLas(+)/(−) ACP; more proteins were downregulated (202) in CLas(+) adult ACP than upregulated (154) (table 1; electronic supplementary material, table S1). By contrast, only 81 proteins (53 CLas upregulated, 28 CLas downregulated) were differentially expressed between CLas(+)/(−) nymphs (table 1; electronic supplementary material, table S2). A greater than 100-fold increase in spectral counts was observed in CLas(+)/(−) adult ACP samples for two proteins, haemocyanin 2 and an asparagine/methionine-rich protein of unknown function, a fold difference far exceeding that calculated for any proteins downregulated in CLas(+) ACP, or any differentially expressed nymphal stage proteins (table 2; electronic supplementary material, tables S1 and S2).

**Table 1.** Number of proteins differentially expressed between nymph and adult populations of CLas(+) and CLas(−) ACP.

	adult CLas(+/−)	nymph CLas(+/−)	CLas(+) adult/nymph	CLas(−) adult/nymph
upregulated	154	53	830	790
downregulated	202	28	1328	1322

**Table 2.** Spectral count data for differentially expressed ACP proteins discussed in this paper. Average spectral count calculated from three biological replicates analysed from each sample category. Statistical analysis using Fisher's exact test (*p*-value < 0.05 with Hochberg–Benjamini multiple testing correction) was used to determine significance of spectral count differences between categories.

		nymph		adult	
protein description	GenBank ID	CLas(−)	CLas(+)	CLas(−)	CLas(+)
transferrin 1	XP_008470528.1	0	0	13	48
transferrin 2	XP_008470513.1	8	8	74	370
mucin 5AC	XP_008474732.1	10	10	3.3	2.6
cuticle protein 21	XP_008478819.1	211	443	34	24
haemocyanin 1	XP_008477906.1	210	494	196	1900
haemocyanin 2	XP_008477908.1	340	602	1.7	216
haemocyanin 3	XP_008477907.1	23	46	3.6	24
unknown asparagine/methionine-rich protein	XP_008486634.1	1	0.7	5.7	897
unknown choline dehydrogenase domain-containing protein	XP_008484178.1	26	13	0.6	1.1

Far more proteins (more than 2000) were differentially expressed between nymph and adult ACP than between CLas(+)/(−) ACP from either developmental stage (table 1; electronic supplementary material, tables S3 and S4). Developmental changes in the ACP impact the efficacy of CLas transmission, with pathogen acquisition in the nymphal stage required for efficient adult transmission. Mucin 5AC, a secreted protein which affects bacterial adhesion and colonization, has been shown to play a central role in host–microbe interactions [32] and was significantly upregulated in nymph ACP compared with adults (table 2). Two transferrin proteins, predicted to function in the insect immune response by sequestering iron to limit bacterial access to this critical nutrient, were found at low or undetectable levels in nymphs, while in adults these proteins were detected at high levels and were found to be upregulated in response to CLas (table 2). The cuticle protein 21 (XP_008478819.1) is upregulated in CLas(+) versus CLas(−) nymph ACP samples, and is also highly upregulated in nymphal ACP compared with adults (electronic supplementary material, tables S2–S4).

### 3.2. Differential expression of endosymbiont proteins

**Figure 1.**
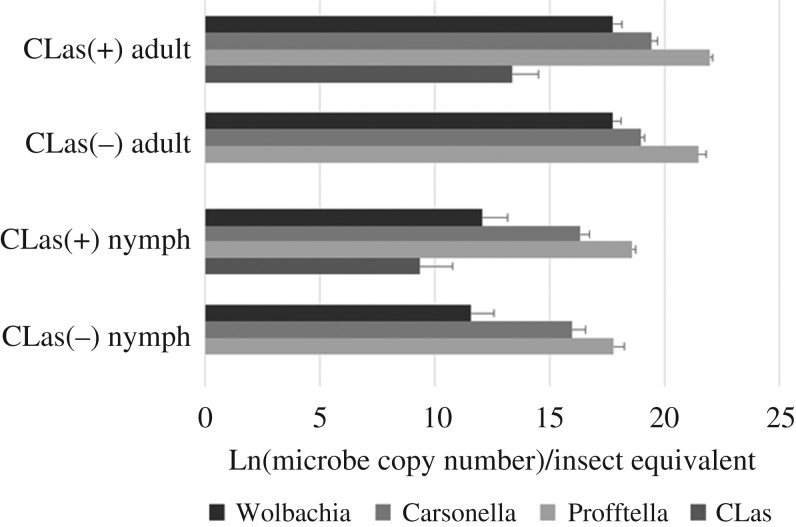
Titre of CLas, Profftella, Carsonella and Wolbachia for nymph and adult ACP estimated by qPCR.

The differentially expressed proteins identified in ACP samples included proteins derived from microbial endosymbionts of the ACP, including ‘*Candidatus* Profftella armatura’, ‘*Candidatus* Carsonella ruddii’ and Wolbachia. Several endosymbiont proteins were differentially expressed between CLas(+)/(−) ACP samples—one Carsonella protein and seven Profftella proteins were upregulated in CLas(+)/(−) adults, and one Profftella protein was downregulated in CLas(+)/(−) nymphs (table 3; electronic supplementary material, tables S1 and S2). Significantly more endosymbiont proteins were differentially expressed between nymph and adult ACP samples than between CLas(+)/(−) samples within either developmental stage (table 2; electronic supplementary material, tables S1–S4). Among the differentially expressed endosymbiont proteins, all Wolbachia proteins and the majority of Profftella proteins were more abundant in adult compared with nymph ACP, while the majority of Carsonella proteins were more abundant in nymph compared with adult ACP. Based on the endosymbiont proteome observations, we hypothesized that the titre of the bacterial symbionts varies between adults and nymphs, as has previously been reported [27]. CLas and endosymbiont titre were estimated by qPCR analysis of DNA purified from ACP of the four conditions considered in this experiment (CLas(+)/(−) nymphs and CLas(+)/(−) adults). No significant difference was found in endosymbiont titre between CLas(+)/(−) ACP of the same developmental stage, and titre of CLas and all three endosymbionts was significantly higher in adults compared with nymphs (figure 1).

**Table 3.** Number of endosymbiont proteins differentially expressed between nymph and adult populations of CLas(+) and CLas(−) ACP.

		adult CLas(+)/(−)	nymph CLas(+)/(−)	CLas(+) adult/nymph	CLas(−) adult/nymph
upregulated	Profftella	7	0	53	38
	Carsonella	1	0	2	0
	Wolbachia	0	0	16	16
downregulated	Profftella	0	1	3	3
	Carsonella	0	0	13	21
	Wolbachia	0	0	0	0

### 3.3. Asian citrus psyllid haemocyanin domain-containing proteins

**Figure 2.**
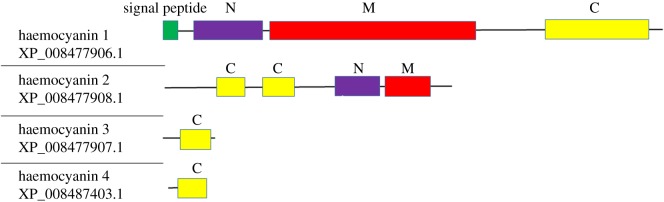
Protein domain map for the four predicted haemocyanin proteins from the ACP, including predicted signal peptide and haemocyanin N, M (oxygen binding) and C (Ig-like) domains.

One of the most highly expressed proteins in CLas(+) adult ACP is predicted by domain analysis to be a haemocyanin (XP_008477906.1, referred to here as haemocyanin 1), an oxygen transport protein found exclusively in arthropods and molluscs. While domain analysis reveals this protein to contain three haemocyanin domains (N, M and C), the GenBank protein name is given as Allergen Cr-PI-like, based on orthology to a cockroach protein characterized for its allergenic properties in humans. ACP haemocyanin 1 has no significant similarity to any hemipteran proteins in public databases. Blastp analysis was conducted using haemocyanin 1 as the query sequence to determine whether additional haemocyanin proteins have been predicted from automated annotation of the ACP genome. The top three resulting ACP protein hits are predicted to contain at least one haemocyanin domain, although none of these proteins are annotated as haemocyanins (figure 2). Quantification based on spectral counting reveals that more peptides derived from haemocyanin 1 protein were detected in CLas(+) adult ACP samples than were peptides from any protein other than an actin protein. More peptides from all three predicted haemocyanin proteins were observed in CLas(+) compared with CLas(−) adults and nymphs (table 2). An 18 amino acid secretion signal is predicted from analysis of haemocyanin 1 by SignalP (http://www.cbs.dtu.dk/services/SignalP/), while haemocyanins 2–4 are not predicted to contain a signal peptide (figure 2).

### 3.4. Correlation of haemocyanin expression with Asian citrus psyllid colour

**Figure 3.**
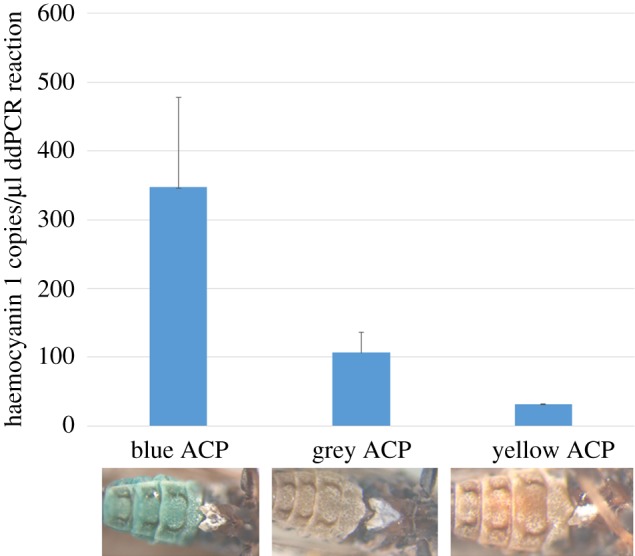
Haemocyanin 1 expression quantification using digital drop PCR. Expression of haemocyanin was found to be more than threefold higher in blue compared with grey ACP colour morphs. Average haemocyanin copy number per microlitre ddPCR reaction plus standard error is shown (*n* = 3, *t*-test *p*-value < 0.1). Haemocyanin expression was lower in yellow ACP than in blue or grey ACP; expression was detected near the limit of instrument detection (32 copies) in one yellow ACP RNA sample, but expression was below the limit of detection in other yellow replicates. Light microscopy images of ventral abdomen of blue, grey and yellow ACP morphs are shown.

Haemocyanin binds copper to transport oxygen, and this oxygenated copper confers a blue colour to the haemolymph of arthropods such as the horseshoe crab (*Limulus polyphemus*), which contains high levels of haemocyanin [33]. ACP exhibit at least three different colour morphs: blue, grey and yellow [34]. Haemocyanin 1 expression was compared among different ACP colour morphs, to evaluate whether insect abdominal colour is correlated with haemocyanin expression. Adult ACP were collected from healthy citron plants and sorted according to abdominal colour (blue, grey and yellow). RNA was isolated from 10 ACP of each colour, and following cDNA synthesis, digital drop PCR was used for quantification of haemocyanin copy number. Haemocyanin expression was found to be more than threefold higher in blue than grey ACP (figure 3). Haemocyanin expression in yellow morph samples was above the limit of detection in only one biological replicate; in this sample, haemocyanin 1 expression was 50% below levels observed in all grey samples.

### 3.5. Protein interaction reporter analysis of *Candidatus* Liberibacter asiaticus(+) Asian citrus psyllid

Protein interaction reporter (PIR) technology was used to identify protein interactions in CLas(+) ACP samples. A total of 733 unique cross-linked peptide pairs were identified—in 712 of these cross-links, both peptides were predicted to be derived from ACP proteins, while 21 cross-linked peptide pairs included at least one peptide derived from CLas or ACP endosymbiont proteins. Peptides derived from ACP haemocyanin 1 were involved in 33 of these interactions—22 cross-links were identified between two peptides both predicted to be derived from haemocyanin 1, while an additional 11 cross-links consisted of a haemocyanin 1 peptide cross-linked to a peptide derived from another protein. Ten of these cross-links are between haemocyanin 1 and a peptide from one of seven ACP proteins, and one cross-link is between haemocyanin 1 and the CLas coenzyme A (CoA) biosynthetic enzyme phosphopantothenoylcysteine decarboxylase/phosphopantothenate synthase (EXU77773.1) (table 4). The cross-links identified between haemocyanin 1 peptides may be either intermolecular cross-links joining separate proteins into oligomers, or intramolecular cross-links between neighbouring regions of an individual protein. One unambiguous homodimer was identified, in which the cross-link was formed between the lysines on two identical haemocyanin 1 peptides, providing support for this region of the protein as an oligomerization interface. Two cross-links were identified between peptides from haemocyanin 1 and a peptide derived from haemocyanin 2, suggesting that haemocyanin may form hetero-oligomers in the ACP. Spectral annotation of all cross-links discussed in this paper is provided in electronic supplementary material, table S5.

**Table 4.** Parent proteins of peptides found cross-linked to haemocyanin 1 peptides. Cross-linked peptide sequences and location of the cross-linked lysine residues within parent proteins are given.

GenBank ID	protein description	haemocyanin 1 peptide	cross-linked peptide
EXU77773.1	phosphopantothenoylcysteine decarboxylase/synthetase (CLas)	LNHK^794^SFNYR	RK^296^DIGDTMR
XP_008477908.1	haemocyanin 2 (ACP)	FNQNGK^825^PLNAEQQR	SNIFPFDRPLK^212^QQNEFNTPNVNQNNAFQQYFCAR
XP_008486634.1	unknown asparagine/ methionine-rich protein (ACP)	LNHK^794^SFNYR	MNK^3^MNR
XP_008486634.1	unknown asparagine/ methionine-rich protein (ACP)	LNHK^794^SFNYR	MNNMNNK^122^MNMMDNNMNR
XP_008481243.1	transcription factor E2F4 (ACP)	LNHK^794^SFNYR	YAAENLEVK^45^QKR
XP_008475271.1	cytochrome P450 6a2 (ACP)	LNHK^794^SFNYR	DSYK^218^ANPSYR
XP_008469921.1	PDZ domain protein (ACP)	NYK^785^AFQHR	MSEDK^5^TPVVR
XP_008487791.1	Vigilin (ACP)	NYK^785^AFQHR	FPPQESKSDK^243^
XP_008485042.1	5′-AMP-activated protein kinase (ACP)	LNHK^794^SFNYR	DFYAASTSTPPCSPSGDPVK^403^THPER

One of the peptides cross-linked to haemocyanin 1 is derived from an ACP protein (XP_008486634.1) which is also highly upregulated by CLas, with a greater than 100-fold increase in spectral counts between CLas(+)/(−) adult ACP samples (table 2). This protein has no significant similarity to any predicted protein in GenBank, and its amino acid composition is heavily skewed towards asparagine (approx. 50%) and methionine (approx. 30%), with a predicted N-glycosylation site at its C-terminus (http://www.imtech.res.in/raghava/glycoep/submit.html). The complementary proteomics approaches of PIR and spectral counting provide independent lines of evidence suggesting that these two proteins are members of an interaction network subject to coordinated regulation in the insect in response to CLas infection.

**Figure 4.**
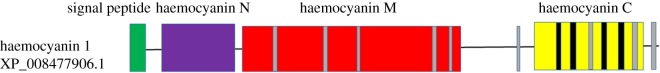
Protein domain map for haemocyanin 1 indicating cross-linked amino acid residues. Using PIR, a total of 17 cross-linked amino acid residues on the haemocyanin protein were identified. The four black bars in the haemocyanin C domain represent the locations of the cross-links discovered between haemocyanin 1 and other proteins. Grey and black bars indicate the location of haemocyanin 1–haemocyanin 1 cross-links discovered.

The CLas protein found to physically interact with ACP haemocyanin 1, phosphopantothenoylcysteine synthetase/decarboxylase, catalyses the second step in the conversion of pantothenate to CoA. Furthermore, a peptide from the CLas enzyme catalysing the first step in CoA biosynthesis, pantothenate kinase, was found to be cross-linked to an ACP myosin-derived peptide (table 5). This vitamin to coenzyme conversion plays a critical role in bacterial metabolic regulation. In the development of antibiotics against human microbial pathogens, enzymes in this pathway have been targeted for inhibition [35]. The discovery of cross-links between ACP proteins and CLas enzymes catalysing sequential steps of an essential microbial metabolic pathway suggests that these interactions may represent an interface for regulation or inhibition of CLas by the ACP. The haemocyanin C (Ig-like) domain is the site of all intermolecular cross-links between haemocyanin 1 and other ACP or CLas proteins, while cross-links between two haemocyanin 1 peptides were found in both the haemocyanin M (oxygen containing) and haemocyanin C domains (figure 4).

**Table 5.** Cross-linked peptides representing interactions between microbe and ACP proteins discussed in this paper. Parent protein IDs, peptide sequences and locations of cross-linked lysine residues within parent proteins are given.

cross-linked microbe protein GenBank ID; description	cross-linked ACP protein GenBank ID; description	microbe peptide sequence	ACP peptide sequence
EXU78401.1; CLas pantothenate kinase	XP_008475404.1; myosin heavy chain	ADLILSKGEDHSVK^304^TIK	LGK^116^IVGWMQSYMR
EXU77773.1; CLas phosphopantothenoylcysteine decarboxylase/synthetase	XP_008477906.1; haemocyanin 1	RK^296^DIGDTMR	LNHK^794^SFNYR
YP_008350653.1; Carsonella imidazoleglycerol phosphate synthase, cyclase subunit	XP_008484178.1; unknown choline dehydrogenase domain-containing	FLK^232^ASYLK	YPYDTLLFPNANLK^1482^ER
AGS06534.1; Carsonella anthranilate synthase component I	XP_008475404.1; myosin heavy chain	IK^144^FFVIK	MVYPDFK^54^LR

**Figure 5.**
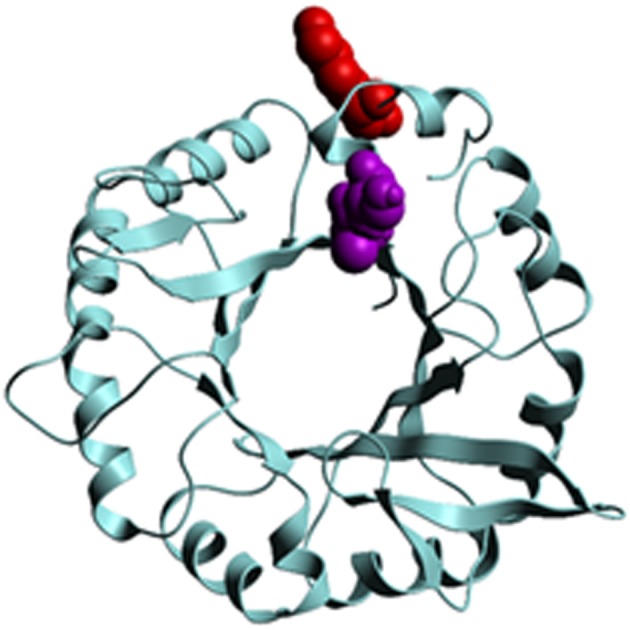
Ribbon diagram depicting predicted three-dimensional structure of Carsonella IGPS. Three-dimensional structure was generated by Molsoft (http://www.molsoft.com/). Cross-linked residue (K_232_) is depicted in red, and glycerol phosphate substrate binding site (A_223_S_224_) is depicted in purple.

Nutritional interactions between hemipterans and their microbial endosymbionts enable these insects to subsist on the unbalanced diet of plant phloem sap [21,22,25]. Two cross-links were discovered between Carsonella amino acid biosynthesis proteins and ACP proteins. The histidine biosynthetic enzyme imidazole glycerol phosphate synthase (IGPS) was found to be cross-linked to a choline dehydrogenase domain-containing ACP protein of unknown function, and the tryptophan biosynthetic enzyme anthranilate synthase was found cross-linked to a peptide derived from an ACP myosin protein (table 5). The predicted interaction site on IGPS is adjacent to the predicted glycerol phosphate substrate-binding site [36] (figure 5). IGPS is subject to allosteric regulation [36], and its interaction with an ACP protein proximal to this substrate-binding site may have a direct or indirect effect on IGPS function. The unknown ACP protein (XP_008484178.1) interacting with IGPS has a predicted size greater than 3000 amino acids, of which a C terminal choline dehydrogenase domain is the only region with significant similarity to other proteins in GenBank. IGPS functions at the interface of histidine and purine biosynthesis: one product, imidazole glycerol phosphate (IGP), is converted into histidine, while the second product, 5-aminoimidazole-4-carboxamide-1-beta-d-ribofuranosyl 5′-monophosphate (AICAR) feeds into the de novo purine biosynthesis pathway. AICAR has been reported to play a role in the regulation of choline metabolism [37], and the interaction of IGPS with the choline dehydrogenase domain-containing protein may represent a metabolic regulatory interface between Carsonella and the ACP. The downregulation of Carsonella proteins during the transition from nymph to adult ACP may indicate a greater role for Carsonella in nymphal metabolism—following the same trend, the peptides from the ACP choline dehydrogenase protein cross-linked to Carsonella IGPS were found at greater than 10-fold higher levels in nymph compared with adult ACP (table 2).

## 4. Discussion

Developmental changes in the ACP impact the ability of the citrus greening insect vector to transmit CLas, with nymphal acquisition of the pathogen required for optimal transmission [9,10]. This study has identified insect proteins differentially expressed between nymph and adult insects that support the hypothesis that nymphs offer a relatively immunopermissive environment facilitating pathogen acquisition and colonization. Two transferrin proteins predicted to contribute to insect defence against bacteria by sequestering iron from invading pathogens were found expressed in adults at high levels, while the same proteins were undetected or found at significantly lower levels in nymphs. In addition, developmental changes in mucin expression may contribute to the establishment of an environment in the nymphal ACP gut more amenable to bacterial acquisition. Upregulation of mucin 5AC has been associated with increased binding of bacterial pathogens to gut tissues in animal pathosystems [32], suggesting that the elevated expression of this protein in nymph ACP may contribute to a gut environment more supportive of CLas acquisition than that found in adults. Peptides from mucin 5AC were identified during characterization of the ACP gut proteome, supporting the hypothesis that this protein functions in the gut [38]. Mucin 5AC belongs to the class of gel-forming mucins, which are wholly secreted into the extracellular space, and decreased production of this protein in adults may inhibit bacterial adhesion to the insect gut, leading to reduced pathogen acquisition.

In this study, CLas(+) ACP were reared on CLas-infected citrus and CLas(−) ACP are reared on healthy citrus, and the differences between these two insect populations include direct effects related to ingestion of the pathogen in infected phloem, and indirect effects related to host plant changes in response to CLas infection. ACP proteins which are upregulated or downregulated in response to CLas, or which physically interact with CLas proteins, are candidate targets for development of novel agents and strategies for control of transmission of the citrus greening pathogen by its insect vector. An ACP haemocyanin protein was identified in this study to be highly upregulated in CLas-exposed insects and to physically interact with a critical CLas metabolic enzyme, suggesting that this protein may play a central role in this pathosystem. Haemocyanins are respiratory proteins of molluscs and arthropods with conserved histidine residues forming a coordination complex with copper ions binding oxygen for transport in the haemolymph [39]. Haemocyanins are believed to have evolved from oxygen-binding tyrosinase enzymes, which participate in melanin biosynthesis and have documented roles in insect defence, including parasite encapsulation [40]. Haemocyanins were determined to be the most abundant haemolymph proteins in the arthropod *L. polyphemus*, comprising 90% of total haemolymph protein measured [39]. The role of haemocyanin in arthropod innate immunity has been documented, including the conversion of haemocyanin into prophenoloxidase [41]. An antimicrobial peptide derived from the C terminus of crayfish haemocyanin was shown to inhibit the growth of both gram-negative and gram-positive bacteria [42]. Haemocyanins are extracellular proteins assembling complex oligomers, including structures consisting of multiple hexamers [39].

The discovery in the ACP of a haemocyanin protein containing a copper-containing domain capable of oxygen transport is unexpected, as no functional haemocyanins have previously been identified in hemipteran insects [43,44]. The respiratory physiology of terrestrial insects is characterized by a well-developed tracheal system connecting inner tissues with the atmosphere, allowing effective transport of oxygen independent of the involvement of respiratory proteins [44]. However, there are examples of insects that use either haemoglobin [45,46] or haemocyanin [47] in oxygen transport, and in the past decade respiratory proteins have been discovered in a wider range of insects than previously predicted [44]. Haemocyanin has a distinct blue coloration when oxygenated, and the haemolymph of organisms that contain large amounts of haemocyanin, such as the keyhole limpet, is bright blue. ACP populations are composed of different colour morphs, including insects with blue coloration visible on their abdomen, which may be derived from haemocyanin. Blue ACP were found to have higher levels of haemocyanin 1 gene expression than grey or yellow morphs (figure 3), lending support to the hypothesis that blue abdominal coloration in the ACP is due at least in part to oxygenated copper associated with this protein. Compared with grey/brown ACP, blue morphs have been found to be capable of longer durations of flight [48], which may be enabled by enhanced oxygen transport capabilities, and to be more insecticide resistant [49]. Additional functional analysis of haemocyanin is warranted to decipher its role at the interface of metabolism and immunity in the ACP.

In the PIR experiments, the protein cross-linker was added to a microbe-enriched fraction prepared from approximately 1000 CLas(+) ACP. This sample preparation method uses Polytron homogenization in a non-lytic buffer and Percoll gradient centrifugation to effectively recover fractions enriched for live microbial cells along with associated insect cells and tissue. Although it is possible that the protein interactions observed in this study are artefacts of this sample preparation method, our experience with PIR suggests that this technology is not effective at capturing transient interactions, and that the number of interactions required to observe a protein interaction are unlikely to occur randomly during sample preparation. Repeating these experiments by adding cross-linker directly to dissected ACP organs, such as the gut, bacteriome and haemolymph, may enrich for CLas and endosymbiont cells or ACP immune components without extensive sample disruption.

The observation of the interaction of ACP haemocyanin 1 with phosphopantothenoylcysteine decarboxylase/phosphopantothenate synthase, a CLas CoA biosynthetic protein, suggests that this fundamental bacterial vitamin metabolism pathway may be targeted by components of the insect immune response. The region of haemocyanin 1 found to interact with the CLas protein is within a C terminal region predicted to have antimicrobial peptide characteristics. Furthermore, a second protein interaction was discovered involving an ACP myosin protein and pantothenate kinase (PanK), the enzyme catalysing the preceding step in CoA biosynthesis. PanK is the rate-limiting enzyme in CoA biosynthesis from pantothenate, and has been the target of antibiotic development in human pathosystems [50].

The interactions between haemocyanin 1 and several other ACP proteins, including an asparagine/methionine-rich protein with greater than 100-fold increased expression in CLas(+) versus CLas(−) ACP, suggests that haemocyanin 1 is part of a multi-component protein interaction network responding in concert to the presence of CLas (table 4). One of the ACP proteins found to interact with haemocyanin 1 contains a PDZ domain, which has documented roles in protein–protein interactions, including linking membrane receptors to the cytoskeleton and propagating signals from outside of the cell to response factors within [51]. Also interacting with ACP haemocyanin 1 is a vigilin protein, which in other systems has been reported to bind to high-density lipoprotein, an abundant component of insect haemolymph [52,53]. The cytochrome p450 6A2 protein interacting with haemocyanin 1 has been found in aphids to play a role in degradation of insecticides and metabolism of insect hormones [54]. The 5′ AMP-activated protein kinase also found to interact with haemocyanin 1 is a sensor of cellular energy status that is activated by metabolic stress [55]. The protein E2F4, a member of a family of transcription factors with roles in cell cycle regulation and apoptosis [56], and haemocyanin 2 are the final ACP proteins found to interact with haemocyanin 1 (table 4). This protein interaction network anchored by haemocyanin proteins is predicted to be a component of the insect vector response to CLas.

In addition to their reported function in oxygen transport and insect immunity, haemocyanin domain-containing proteins with larval or nymphal stage-specific roles in nutrient storage have been described [57]. Termed hexamerins, these larval storage proteins have lost their copper binding function and accumulate to extremely high concentrations in larval or nymphal haemolymph [58]. Hexamerins are believed to serve as a source of amino acids and energy for the development of adult body components during metamorphosis, and these proteins have been incorporated into the pupal cuticle and the fat body [58]. Expression of haemocyanin 2 and 3 is drastically reduced in the developmental transition from nymph to adult in CLas(−) insects, while CLas(+) adults continue to express these proteins at high levels (table 2). This suggests that these proteins have a functional plasticity, and the developmental signal to reduce expression of this nymphal specific protein can be overridden by an immune response trigger which exploits the capabilities of these proteins to participate in the bacterial defence response.

Interactions with microbial endosymbionts play a critical role in the biology of the ACP, and metabolic changes in the Profftella endosymbiont have been associated with CLas infection [12]. Observation of increased titre of all three endosymbionts in adult compared with nymph ACP is consistent with published data showing an increase in endosymbiont titre per insect equivalent during development of the ACP [27]. Increased abundance of a large number of Wolbachia and Profftella proteins in adult compared with nymph ACP may result simply from an increased endosymbiont titre in the adults, rather than developmental stage-specific induction of protein expression. On the other hand, the fact that nearly all differentially expressed Carsonella proteins are more abundant in nymphs compared with adults may indicate that Carsonella metabolism plays a critical role in the biology of ACP nymphs (table 3).

The discovery of protein interactions between the ACP and Carsonella has the potential to reveal mechanistic details of the nutritional symbiosis in this host–microbe relationship. Integrated metabolic pathway analysis of the Hackberry petiole gall psyllid (*Pachypsylla venusta*) and its Carsonella symbiont has been used to support the hypothesis of shared amino acid metabolism [59]. The physical interaction reported here between the Carsonella histidine biosynthesis protein IGPS (encoded by the HisH gene) and a choline dehydrogenase domain-containing ACP protein, represents a potentially critical interface between host and microbe relevant to the nutritional symbiosis. Compelling elements of this ACP--Carsonella protein interaction include the location of the cross-linking site on the substrate binding site of the IGPS protein, with potential consequences on protein function (figure 5), and the significance of choline to insect metabolism. The role of one of the IGPS products, AICAR, in regulating choline metabolism [37,60] may represent a mechanism for metabolic regulation between host and symbiont. Unlike related strains of Carsonella identified in other insects [61], the Carsonella strain of the ACP retains all genes required to synthesize tryptophan and histidine [22]. The interaction between an ACP myosin protein and Carsonella anthranilate synthase, the rate-limiting enzyme in tryptophan biosynthesis, when taken together with the IGPS cross-link and the gene losses in histidine and tryptophan biosynthetic pathways across Carsonella evolutionary history [61], suggests that these proteins play an important role in this symbiosis and as such may be valuable targets for vector control.

## Supplementary Material

File S1. Supplemental methods: Protein Interaction Reporter (PIR) crosslinking

## Supplementary Material

Table S1. Spectral count data for differentially expressed proteins identified between CLas(+)/(-) adult ACP. Average spectral count calculated from three biological replicates analyzed from each sample category. Statistical analysis using Fisher's exact test (p-value<0.05 with Hochberg-Benjamini multiple testing correction) was used to determine significance of spectral count differences between categories

## Supplementary Material

Table S2. Spectral count data for differentially expressed proteins identified between CLas(+)/(-) nymph ACP. Average spectral count calculated from three biological replicates analyzed from each sample category. Statistical analysis using Fisher's exact test (p-value<0.05 with Hochberg-Benjamini multiple testing correction) was used to determine significance of spectral count differences between categories

## Supplementary Material

Table S3. Spectral count data for differentially expressed proteins identified between Adult and Nymph CLas(+) ACP. Average spectral count calculated from three biological replicates analyzed from each sample category. Statistical analysis using Fisher's exact test (p-value<0.05 with Hochberg-Benjamini multiple testing correction) was used to determine significance of spectral count differences between categories

## Supplementary Material

Table S4. Spectral count data for differentially expressed proteins identified between Adult and Nymph CLas(-) ACP. Average spectral count calculated from three biological replicates analyzed from each sample category. Statistical analysis using Fisher's exact test (p-value<0.05 with Hochberg-Benjamini multiple testing correction) was used to determine significance of spectral count differences between categories

## Supplementary Material

Table S5. Annotation of reported PIR crosslinks. Search engine output: react2 data files for each crosslink are listed in the two rightmost columns. React2 files and the corresponding mzXML files have been uploaded to ProteomeXchange as part of this publication.

## Data accessibility

The mass spectrometry proteomics data have been deposited to the ProteomeXchange Consortium (http://proteomecentral.proteomexchange.org) via the PRIDE partner repository [62] with the dataset identifiers: PXD004225, PXD004229, PXD004230, PXD004449, PXD004454.
