# Supplementary material for: Protein interaction networks at the host–microbe interface in Diaphorina citri, the insect vector of the citrus greening pathogen
Source: R Soc Open Sci. 2017 Feb 8;4(2):160545. doi: 10.1098/rsos.160545 (PMC5367280; doi:10.1098/rsos.160545)
Supplement: File S1. Supplemental methods: Protein Interaction Reporter (PIR) crosslinking [file rsos160545supp1.docx]

**File S1.** Supplemental methods: Protein Interaction Reporter (PIR) crosslinking.

Percoll fraction samples enriched for CLas cells were isolated from ~1000 adult ACP feeding on CLas infected citron plants and suspended in a buffered solution consisting of 50 mM Na_2_HPO_4_ and 0.85 % (w/v) NaCl pH 7.4. The chemical cross-linking reaction was carried out using a 10 mM concentration of the PIR cross-linker (Biotin Aspartate Proline-N-hydroxyphthalamide, BDP-NHP) for two hours at room temperature.

Protein was extracted with 8 M urea in 0.1 M Tris-HCl pH 8.0. Disulfide bonds were reduced with 5 mM tris(2-carboxyethyl)phosphine (TCEP) for 30 minutes followed by alkylation by a 45 minute incubation with 10 mM iodoacetamide. The urea concentration was diluted to less than 1 M and proteins were digested with using a 1:200 ratio of trypsin to protein at 37^o^C for 18 hr. Cross-linked peptides were enriched with 100 μL of immobilized monomeric avidin slurry for 30 minutes at room temperature. To remove non-biotin containing peptides, the avidin beads were washed 3 x with 3 mL 100 mM NH_4_HCO_3_ pH 8.0 before eluting the cross-linked peptides by incubating the beads for five minutes each with two 500 uL aliquots of 70% acetonitrile, 30% H20 containing 0.5 % formic acid. The enriched cross-linked peptide sample was then concentrated by vacuum centrifugation and stored at -80oC until LC-MS analysis.

Samples containing PIR cross-linked peptides were analyzed by liquid chromatography mass spectrometry using a Waters NanoAcquity UPLC coupled to a Thermo Velos-FTICR mass spectrometer (1) and a real-time adaptive, targeted mass spectrometry method developed for PIR cross-linked peptides (2). The Velos-FTICR mass spectrometer was operated utilizing ReACT where ions with a charge state of four or greater were selected for high resolution MS^2^ analysis in the ICR cell where an “on-the-fly” check of the observed fragment ion masses against the PIR mass relationship (Mass Precursor = Mass Reporter Ion + Mass Peptide 1 + Mass Peptide 2) is performed. Masses that satisfied the PIR relationship within a tolerance of 20 ppm mass error triggered subsequent low resolution MS3 analyses of the released cross-linked peptide ions.

ReACT generated MS^3^ spectra containing peptide fragmentation information were searched against a database containing both forward and reverse protein sequences for *Diaphorina citri*, ‘*Candidatus* Liberibacter asiaticus’, Wolbachia, Profftella, and Carsonella (66224 total sequences) using Comet (3) (v. 2015.01). Comet search parameters included; a 20 ppm precursor mass tolerance, allowing for -1, 0, +1, +2, or +3 13C offsets, a 1.005 Da fragment ion mass tolerance with 0.4 Da offset, and variable modification of oxidation (15.9949 Da) on Met and the BDP stump mass (197.0324 Da) on Lys, considering only fully tryptic peptide sequences and allowing for up to 3 missed cleavage sites. False discovery of cross-linked peptides was addressed by searching a concatenated database containing forward and reverse protein sequences as previously described (4). Briefly, cross-linked peptide sequences were reported for cases where both peptide sequences contained an internal Lys residue modified by the cross-linker (197.0324 Da) and were assigned at <5% false discovery rate using a forward/reverse database search strategy. The global FDR was estimated to be less than or equal to 1.8%, and was measured by allowing reverse peptide sequences that pass the 5% FDR threshold at the identification stage to be mapped to PIR relationships and taking the ratio of decoy cross-linked peptide pairs (either one or two reverse peptide sequences) to the total number of cross-linked peptide pairs.

**References File S1**

1. Weisbrod CR, Hoopmann MR, Senko MW, Bruce JE. Performance evaluation of a dual linear ion trap-Fourier transform ion cyclotron resonance mass spectrometer for proteomics research. J Proteomics. 2013;88:109-19.

2. Weisbrod CR, Chavez JD, Eng JK, Yang L, Zheng CX, Bruce JE. In Vivo Protein Interaction Network Identified with a Novel Real-Time Cross-Linked Peptide Identification Strategy. J Proteome Res. 2013;12(4):1569-79.

3. Eng JK, Hoopmann MR, Jahan TA, Egertson JD, Noble WS, MacCoss MJ. A Deeper Look into Comet-Implementation and Features. J Am Soc Mass Spectr. 2015;26(11):1865-74.

4. Chavez JD, Schweppe DK, Eng JK, Zheng CX, Taipale A, Zhang YY, et al. Quantitative interactome analysis reveals a chemoresistant edgotype. Nat Commun. 2015;6.
